# Fatigue behavior and translucency of lithium disilicate ceramic: effect of glazing application

**DOI:** 10.1590/0103-644020266769

**Published:** 2026-04-10

**Authors:** Bibiana Vogel Peres Riesgo, Luiza Ribeiro, Pablo Machado Soares, Luiza Freitas Brum Souza, Luiz Felipe Valandro, Gabriel Kalil Rocha Pereira, Liliana Gressler May

**Affiliations:** 1 MSciD and PhD Post-Graduate Program in Oral Science, Faculty of Dentistry, Federal University of Santa Maria (UFSM), Santa Maria, Rio Grande do Sul State, Brazil; 2 Faculty of Dentistry, Federal University of Santa Maria (UFSM), Santa Maria, Rio Grande do Sul State, Brazil

**Keywords:** CAD/CAM, CEREC Tessera ceramic, Roughness, Fatigue resistance, Translucency properties

## Abstract

Este estudo avaliou o efeito de diferentes protocolos de aplicação de glaze sobre a rugosidade superficial, a translucidez e o comportamento à fadiga de uma cerâmica à base de dissilicato de lítio (CEREC Tessera, Dentsply, Alemanha). Discos (cor A2 HT; Ø = 10 mm; espessura = 1 mm) foram confeccionados e distribuídos aleatoriamente em três grupos (n = 7 para rugosidade e translucidez; n = 12 para fadiga): CTRL (apenas cristalização), CCG (cristalização e glaze em um único ciclo) e C+G (cristalização seguida de um ciclo adicional de glaze). A rugosidade superficial (Ra, Rz) foi mensurada com um perfilômetro de contato, e a translucidez (TP_00_) foi calculada a partir das coordenadas de cor CIEDE2000. O ΔTP entre os grupos também foi determinado. Os espécimes foram cimentados a discos de resina epóxi reforçada por fibras de vidro e submetidos a ensaio de fadiga cíclica sob imersão em água até a fratura. ANOVA de uma via e testes post-hoc de Tukey foram utilizados para rugosidade e translucidez, e Kaplan-Meier com teste de Mantel-Cox para fadiga. Um espécime fraturado de cada grupo foi analisado por MEV para observar a iniciação e propagação das trincas. A aplicação do glaze aumentou a rugosidade superficial, sendo CCG e C+G mais rugosos que CTRL (p > 0,05). Os valores de TP_00_ variaram de 27,12 (C+G) a 28,33 (CCG), sem diferenças significativas. Os valores de ΔTP (0,30 para CCG e 0,91 para C+G) ficaram abaixo do limiar de perceptibilidade. O desempenho à fadiga diferiu entre os grupos (p < 0,05), com C+G apresentando maior número médio de ciclos até a falha em comparação com CTRL e CCG, embora a carga de falha não tenha diferido significativamente. A análise em MEV revelou trincas iniciando na superfície de cimentação sob tensão e se propagando em direção à superfície oclusal.

## Abstract (en)

This study evaluated the effect of glaze application protocols on surface roughness, translucency, and fatigue behavior of a lithium disilicate-based ceramic (CEREC Tessera, Dentsply, Germany). Discs (A2 HT shade; Ø = 10 mm; thickness = 1 mm) were fabricated and randomly assigned to three groups (n = 7 for roughness and translucency; n = 12 for fatigue): CTRL (crystallization only), CCG (combined crystallization/glaze firing), and C+G (crystallization followed by additional glaze firing). Surface roughness (Ra, Rz) was measured using a contact profilometer, and translucency (TP_00_) was calculated from CIEDE2000 color coordinates. The ΔTP between groups was also determined. Specimens were cemented to glass fiber reinforced epoxy resin discs and subjected to cyclic fatigue testing under water immersion until failure. One-way ANOVA and Tukey’s post-hoc tests were used for roughness and translucency, and Kaplan-Meier with Mantel-Cox for fatigue. One fractured specimen per group was examined by SEM to assess crack initiation and propagation. Glazing increased surface roughness, as CCG and C+G showed higher Ra and Rz values compared to CTRL (p > 0.05). TP_00_ ranged from 27.12 (C+G) to 28.33 (CCG), with no significant differences. ΔTP values (0.30 for CCG, 0.91 for C+G) were below the perceptibility threshold. Fatigue performance differed among groups (p < 0.05), with C+G showing higher mean cycles to failure compared to CTRL and CCG, although fatigue failure load did not differ significantly. SEM analysis revealed fractures initiating at the cementation surface under tensile stress and propagating toward the occlusal surface.

**Figure:**
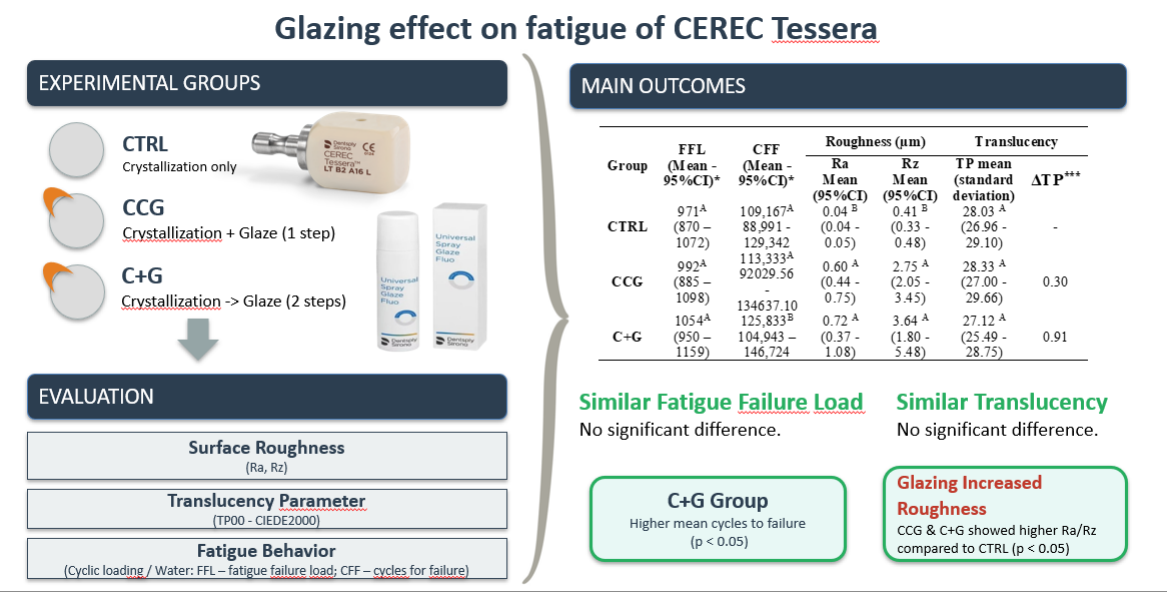

## Introduction

Lithium disilicate glass ceramics (LD) have gained significant importance as an essential restorative material in the field of prosthetic dentistry^1^. This ceramic presents excellent optical characteristics and respectable strengths, depicted by its microstructure that contains both vitreous and crystalline content^2^. Thus, these aspects make lithium disilicate suitable for time-efficient digital fabrication of dental crowns for both posterior and anterior regions through computer-aided design/computer-aided manufacturing systems (CAD/CAM)^3^. 

The first released CAD/CAM LD ceramic blocks were composed of 40% metasilicate crystals and LD nuclei embedded in a glassy phase. The blocks are supplied in a partially crystalline intermediate phase (blue state) to facilitate machining^4^, and the final crystallization stage occurs in a ceramic furnace wherein metasilicates dissolve, and LD undergoes crystallization. The resulting material comprises 70% fine-grain lithium disilicate crystals within a glassy matrix, delivering sufficient mechanical strength^5^.

After the patent expiration of the first lithium disilicate-based ceramic, new formulations were developed, including CEREC Tessera (Dentsply Sirona, Bensheim, Germany). This material contains lithium aluminum silicate crystals, known as virgilite^6^, which are embedded in a vitreous matrix^7^. According to recent compositional analyses, the investigated ceramic comprises approximately 90% lithium disilicate crystals (0.5 μm) and 5% virgilite (0.2 - 0.3 μm) by volume^8^. However, the presence of virgilite remains controversial. Some authors, such as Hurle et al.^9^, suggest that this phase may actually correspond to a solid solution of stuffed quartz rather than true virgilite. These differences in crystal structure and phase composition may influence the mechanical properties and translucency of the ceramic, as well as its response to firing cycles. In terms of performance, Balladares et al.^10^ reported that crystallized CEREC Tessera exhibits high fracture resistance (~ 437 N), comparable to that of IPS e.max CAD (~ 435 N), indicating its suitability for clinical use.

Machining can lead to surface defects, which may concentrate stresses and propagate to failures in ceramic specimens^11^, reducing flexural strength and fatigue resistance^12^. Thus, proper finishing techniques, such as glaze firing and polishing, are indicated to improve the surface topography^13^. However, results show that glaze firing cycles may not effectively eliminate machining-induced defects^14^, and also sometimes negatively impact mechanical strength in vitreous ceramics^12^ ^,^ ^13^. Besides, Abuhammoud et al.^15^ reported that glazed CEREC Tessera presented higher surface roughness (Ra ≈ 52 μm) compared to polished (Ra ≈ 15 - 25 μm), and to glazed IPS e.max CAD (Ra ≈ 10 μm). These findings suggest that while the manufacturer’s rapid firing cycle may offer time efficiency, it might compromise surface quality depending on the applied finishing protocol. There is still no consensus regarding the most effective surface-finishing protocol for this material, which may influence both optical and mechanical performance in clinical applications. This uncertainty has important clinical implications, as an inadequate surface-finishing protocol can increase surface roughness, favoring plaque accumulation and micro-crack initiation that may lead to premature chipping, fractures, esthetic deterioration, and ultimately restoration replacement^16^.

As mentioned, CEREC Tessera blocks are supplied partially crystallized, requiring a mandatory rapid firing cycle for both matrix and glaze (4 minutes and 30 seconds). The manufacturer emphasizes that combining glaze firing with crystallization is essential to achieving the material’s final strength. The so-called healing effect refers to the reduction or sealing of machining-induced surface defects and microcracks through viscous flow during the refiring process, which can reduce stress concentration and limit crack propagation, thereby enhancing fatigue resistance. However, this same process may also modify the glass-crystal interface, influencing light scattering and consequently the translucency of the material^17^. Therefore, further studies are necessary to clarify the ideal glaze application protocol to improve the long-term mechanical and optical performance of lithium disilicate restorations.

Considering the aforementioned context, the goal of this *in vitro* study was to evaluate the effect of the application of glaze spray and glaze firing (combined or not with crystallization) on the surface roughness, translucency, and fatigue behavior of CEREC Tessera. The null hypotheses were that the glaze application protocol would not affect^1^ surface roughness,^2^ translucency, and^3^ fatigue performance of the restorative set.

## Material and methods

### Study design

Discs of a lithium disilicate ceramic (CEREC Tessera, Dentsply Sirona) in A2 HT shade were obtained (Ø = 10 mm and 1 mm in thickness) from prefabricated blocks. For the mechanical test, glass fiber reinforced epoxy resin discs were also obtained (Ø = 10 mm and 2.5 mm in thickness). Commercial brands, compositions, and lot details of each material are provided in Table 1. The experimental design is depicted in Table 2.

**Table 1. Materials:** used in the study and description of their commercial names, manufacturers, compositions and batch numbers.

Ceramic material	Commercial name / manufacturer	Composition	Batch number
Lithium disilicate-based ceramic	CEREC Tessera HT shade A2, Dentsply Sirona, Hanau-Wolfgang, Germany	Li_2_Si_2_O_5_ (90.0 wg%) Li_3_PO_4_ (5.0 wg%) Li_0.5_Al_0.5_Si_2.5_O_6_ (virgilite): 5%	16013774
Spray glaze	Universal Spray Glaze, Dentsply Sirona, Hanau-Wolfgang, Germany	Silicate glass, isopropyl alcohol, isobutane, propellant	A0952
5% hydrofluoric acid	Condac Porcelana, FGM, Joinville, Brazil	<5% hydrofluoric acid	230822
10% hydrofluoric acid	Condac Porcelana, FGM, Joinville, Brazil	<10% hydrofluoric acid	300522
Ceramic primer coupling agent	Monobond N, Ivoclar Vivadent, Schaan, Liechtenstein	Alcohol solution of silane methacrylate, phosphoric acid methacrylate and sulphide methacrylate	Z02M0Y
Dual-cure resin cement	Multilink N, Ivoclar Vivadent Schaan, Liechtenstein	Silicate glass, ytterbium trifluoride, highly dispersed silica, catalysts and stabilizer, pigments	Z00JPZ
Primer	A and B Multilink Primer, Ivoclar Vivadent, Schaan, Liechtenstein	A Primer: water, initiators; B primer: phosphonic acid acrylate, hydroxyethyl methacrylate, methacrylate mod. polyacrylic acid, stabilizer	A primer: Z02L2B B primer: Z02SM9
Epoxy resin	Protec, São Paulo, Brazil	Continuous filament woven fiberglass bonded with epoxy resin	-

* The chemical composition is described according to the manufacturer's information.

**Table 2:** Experimental group

Group	Treatment	Tests performed
CTRL	Only LD Crystallization firing*	Surface roughness (n=7) Translucency (TP_00_) (n=7) Cyclic Fatigue (n=12) Weibull analysis (n=12)
CCG	Application of a spray glaze layer; Crystallization/glazing combined firing*	
C+G**	Crystallization firing, followed by the application of a glaze spray layer, and additional glaze firing*/**	

Heat treatments followed the recommendations for the VITA VACUMAT 6000 MP furnace:

*Firing Protocol: Closing: 2 min. Pre-heating temperature: 400°C. Heating rate: 55°C/min. Firing temperature: 760°C. Holding time: 2 min. Vacuum 1 and 2: -. Long-term cooling: 0°C/min.

**Firing protocol 2: Closing: 1 min. Pre-heating temperature: 400°C. Heating rate: 55°C/min. Firing temperature: 750°C. Holding time: 2 min. Vacuum 1 and 2: -. Long-term cooling: 500/0:10°C/min.

### Specimen preparation

The CEREC Tessera blocks were shaped into a cylindrical format (10 mm in diameter) under constant cooling using a coarse grinding diamond disc (Coarse Grinding Discs in green color - grit 240 μm, Buehler) on a polishing machine (Ecomet/Automet 250, Buehler). Metal references of 10 mm in diameter were affixed on both sides of the block to guide this procedure. After that, the cylinders were cut (IsoMet 1000, Buehler) under water-cooling into discs with a thickness of 1.1 mm. Both surfaces of all discs were ground and polished (EcoMet/AutoMet 250, Buehler) using Silica carbide (SiC) abrasive papers (grits # 400, # 600, and # 1200, 3M; Sumaré, Brazil) to achieve a final standardized thickness of 1.0 mm and a uniform surface finish. After, they were cleaned in an ultrasonic bath with isopropyl alcohol for 5 minutes.

A total of 12 specimens were fabricated per group (n = 12). The specimens were randomly divided into three groups (Random.org) based on the glaze firing protocol outlined in Table 2: Group 1 (CTRL) underwent only crystallization through thermal treatment; Group 2 (CCG) combined crystallization and glazing firing in a single cycle, in which the lithium disilicate specimens received a spray glaze layer and were fired in one rapid sintering cycle; and Group 3 (C+G) was first subjected to one crystallization firing, followed by a spray glaze layer application and an additional glazing firing. All groups underwent firings using a Vacumat 6000 MP furnace (VITA Zahnfabrik, Bad Sackingen, Germany), following the manufacturer’s guidelines.

For the CCG and C+G groups, the glaze spray (Universal Spray Glaze, Dentsply Sirona, Bensheim, Germany) was applied according to the manufacturer’s instructions. Before application, the bottle was shaken, and a single, uniform jet of glaze was sprayed from approximately 15 cm away, maintaining a perpendicular angle (90°) to the specimen surface. The applied layer was then fired according to the specific thermal protocol of each group. The glaze was applied on the upper surface of the ceramic discs, which was also the surface used for both roughness and translucency evaluations.

### Roughness analysis

After the specimen preparation and crystallization protocols, a surface roughness analysis was conducted (n = 7) using a contact stylus profilometer (Mitutoyo SJ-410, Japan). The parameters Ra (mean surface roughness) and Rz (maximum peak-to-valley height at five regions) were measured, following the ISO 4287: 1997^19^. For each specimen, six measurements, three in each axis (x and y), were taken with a cut-off (λϲ) of 0.8 mm and a sampling length of 4 mm. The mean values of Ra and Rz parameters were calculated for each group for posterior statistical purposes.

### Translucency parameter

The translucency parameter was measured in the CIE Lab* color space^20^, a three-dimensional color space system, using a spectrophotometer (SP60, EX-Rite, Grand Rapids, MI, USA). In this system, L* represents the brightness coordinate (with values ranging from 0 (black) to 100 (white)), while a* and b* denote the chromatic coordinates on the red-green and yellow-blue axes, respectively. The measurements were performed three times on the top surface of each sample (where the glaze is present) for each group (n = 7) against black (L* = 11.98, a* = -0.69, b* = -1.42) and white (L* = 91.24, a* = -0.63, b* = 4.55) backgrounds, and the mean values of each parameter were obtained. The translucency parameter (TP_00_), defined as the color difference on black and white backgrounds, was calculated using the following CIEDE 2000 formula:

$${{TP}_{00}=\left[{\left(\frac{{L^{'}}_{1}-\;{L^{'}}_{2}}{K_{L}S_{L}}\right)}^{2}+{\left(\frac{{C^{'}}_{1}-\;{C^{'}}_{2}\;}{K_{C}S_{C}}\right)}^{2}+\;{\left(\frac{{H^{'}}_{1}-\;{H^{'}}_{2}}{K_{H}S_{H}}\right)}^{2}+R_{T}\;\left(\frac{{C^{'}}_{1}-\;{C^{'}}_{2}}{K_{C}S_{C}}\right)\;\left(\frac{{H^{'}}_{1}-\;{H^{'}}_{2}}{K_{H}S_{H}}\right)\right]}^{\frac{1}{2}}$$

In which: the subscripts "1" and "2" refer to lightness (L′), chroma (C′), and hue (H′) of the specimens over the black and the white backgrounds, respectively. RT represents a rotation function that considers the interaction between chroma and hue differences in the blue region. SL, SC, and SH are weighting functions, while KL, KC, and KH are correction factors adjusted according to experimental conditions (predefined as 1)^20^ ^,^ ^21^. A coupling substance (glycerol, C3H8O3) (Vetec Química Fina Ltda., Rio de Janeiro, Brazil) with a refractive index of 1.47 was used to minimize light diffraction by eliminating the presence of an air layer between the sample and the background^22^. To compare the translucency level between the groups, a perceptibility threshold (ΔTP > 2) described by Lee 2015^23^ was adopted.

### Fabrication of glass fiber reinforced epoxy resin discs

The glass fiber reinforced epoxy resin discs (Protec, São Paulo, Brazil) were made by slicing a 10-mm rod bar in a cutting machine (IsoMet 1000, Buehler) into 2.6 mm-thick and 10 mm-diameter slices. Then, the discs were manually polished on both sides with grit silicon carbide papers (# 400 - and # 1200 - grit) until they achieved their final thickness (2.5 mm) and cleaned in an ultrasonic bath with distilled water for 5 min. 

### Cementation

All ceramic discs underwent ultrasonic cleaning with isopropyl alcohol for 5 minutes before the bonding procedures. The bonding surfaces of all ceramic discs were then etched for 20 seconds with 5% hydrofluoric acid (Condac Porcelana 5%, FGM, Joinville, Brazil), rinsed for 30 seconds with air-water spray, and air-dried. The specimens were subsequently cleaned in an ultrasonic bath with distilled water for 5 minutes to remove any residue from the etching procedure and air-dried. A silane-containing primer (Monobond N, Ivoclar AG) was applied over the etched surface for 60 seconds and then gently air-dried. 

For the glass fiber reinforced epoxy resin discs, the bonding surface was etched with 10% HF for 60 seconds (Condac Porcelana 10%, FGM), rinsed for 30 seconds with air-water spray, and air-dried. After cleaning in an ultrasonic bath with distilled water for 5 minutes, Multilink Primers A and B (Ivoclar AG) were mixed in a 1:1 ratio, brushed on the etched surface for 30 seconds, and air-dried to obtain a thin layer. Dual-curing resin cement (Multilink N, Ivoclar Vivadent) was manipulated as recommended by the manufacturer’s instructions and applied to the center of the ceramic bonding surface. Finally, each ceramic disc was luted to the corresponding analogue disc under a constant load of 2.5 N. Excess resin cement was removed from the margins, and the resin cement was light-activated (Radii-Cal, SDI, Bayswater, Australia, 1200 mW/cm² irradiance) by five exposures of 20 seconds each around the specimen (0°, 90°, 180°, 270°, and on the top surface). The bonded assemblies were then stored in distilled water (37 °C) for 24 hours up to 7 days until fatigue testing.

### Cyclic fatigue test

The restorative set (n = 12) was tested using a cyclic fatigue test approach in a mechanical testing machine (Instron ElectroPlus E3000, Instron, Norwood, USA). All tests were performed in a climate-controlled environment maintained at 20 °C. An adhesive tape (110 μm) was fixed between the piston and the top surface of the sample before testing, in order to prevent surface contact damage^23^. The specimens were positioned on a flat support base, and the load was applied at the top surface of the ceramic with a 40-mm diameter stainless steel hemispheric piston^24^ under a frequency of 20 Hz, with the specimens immersed in distilled water. The initial load was 200 N for 5000 cycles to accommodate the relation between the specimen and the piston. Then, the load was adjusted to 300 N for 10,000 cycles, and incremental steps of 100 N were applied every 10,000 cycles until reaching 700 N for 55,000 cycles. Finally, increments of 50 N were applied until specimen failure, indicated by radial cracks or fractures, detected by transillumination. Then, fatigue failure load (FFL) and number of cycles for failure (CFF) were registered for each specimen. 

### Fractographic analysis

The ceramic fragments were detached from the substrates to allow access to the fractured surfaces. Subsequently, one representative specimen per group was analyzed using Scanning Electron Microscopy (SEM, JSM-6360, JEOL, Tokyo, Japan) at 200× and 500× magnifications to identify the failure origin and describe the characteristic fracture features.

### Data analysis

The statistical analyses for surface roughness, translucency, FFL, and CFF data were performed using SPSS software (IBM SPSS, IBM Corp., Armonk, USA). Data normality was verified using the Shapiro-Wilk test. Since both surface roughness (Ra and Rz) and translucency (TP_00_) data followed a normal distribution (p > 0.05), one-way ANOVA followed by Tukey’s post-hoc test (α = 0.05) was applied for group comparisons. The ΔTP values were determined by calculating the difference between the translucency means of each group.

The fatigue failure load (FFL) in Newtons (N) and the number of cycles to failure (CFF) were subjected to Kaplan-Meier survival analysis with post-hoc Mantel-Cox test (α = 0.05). The survival probability was calculated and tabulated for each stage of the fatigue test. 

Additionally, FFL and CFF data underwent Weibull statistical analysis, using the SuperSMITH Weibull 4.0k-32 software program (Wes Fulton, Torrance, United States) to estimate the mechanical structural reliability (m - Weibull modulus), and their respective 95% confidence interval (CI) values, considering the non-overlapping of CI as an indicator of significant differences among groups.

## Results

Table 3 presents an overview of the study findings, including FFL, CFF, and their respective Weibull modulus, roughness parameters (Ra and Rz), translucency parameters (TP_00_) values for the groups under investigation, and ΔTP. 

**Table 3:** Mean, Weibull modulus for fatigue failure load (FFL), number of cycles until failure (CFF), roughness (Ra and Rz), their respective 95% confidence intervals, translucency parameter (standard deviation) values for the studied materials, and translucency difference.

Group	FFL (Mean - 95%CI)*	Weibull Modulus for FFL (95%CI)**	CFF (Mean - 95%CI)*	Weibull Modulus for CFF (95%CI)**	Roughness (µm)		Translucency	
					Ra Mean (95%CI)	Rz Mean (95%CI)	TP mean (standard deviation)	ΔTP^***^
CTRL	971^A^ (870 - 1072)	6.67^A^ (4.13 - 9.86)	109,167^A^ (88,991 - 129,342)	3.85^A^ (2.38 - 5.72)	0.04^B^ (0.04 - 0.05)	0.41^B^ (0.33 - 0.48)	28.03^A^ (26.96 - 29.10)	-
CCG	992^A^ (885 - 1098)	6.44^A^ (4 - 9.46)	113,333^A^ (92,029 - 134,637)	3.79^A^ (2.34 - 5.60)	0.60^A^ (0.44 - 0.75)	2.75^A^ (2.05 - 3.45)	28.33^A^ (27.00 - 29.66)	0.30
C+G	1054^A^ (950 - 1159)	7.41^A^ (4.6 - 12)	125,833^B^ (104,943 - 146,724)	4.47^A^ (2.72 - 6.70)	0.72^A^ (0.37 - 1.08)	3.64^A^ (1.80 - 5.48)	27.12^A^ (25.49 - 28.75)	0.91

* Different letters in such columns indicate statistical differences depicted by Kaplan-Meier and Mantel-Cox post-hoc tests (α = 0.05).

** Different letters in such columns indicate statistical differences depicted by Weibull analysis using the maximum likelihood estimation method, whereas the absence of overlap of the 95% confidence intervals means statistical differences.

*** The translucency difference (ΔTP) was measured by calculating the difference in translucency level from CCG and C+G when compared to the CTRL (reference group).

Surface roughness parameters (Ra and Rz) revealed notable differences among the groups (ANOVA: Ra p = 0.024; Rz p = 0.027). The CCG (Ra = 0.60; Rz = 2.75) and C+G (Ra = 0.72; Rz = 3.64) groups presented rougher surfaces, while the CTRL was remarkably smoother (Ra = 0.04; Rz = 0.41).

The TP_00_ values regarding translucency varied across the groups, ranging from 27.12 in the C+G group to 28.33 in the CCG group and 28.03 in the *CTRL*, with no statistical difference among the groups (p ≥ 0.05). The ΔTP between CCG and CTRL groups was 0.30, while the ΔTP between C+G and CTRL groups was 0.91, both below the visual perceptibility threshold (ΔTP < 2), indicating clinically imperceptible differences.

FFL values (ranging from 971 N in CTRL to 1054 N in C+G) showed no significant differences among groups (p ≥ 0.05). However, CFF values differed significantly (p < 0.05), with the C+G group exhibiting higher mean cycles to failure(125,833) compared to CTRL (109,167) and CCG (113,333).

For a detailed understanding of the survival probabilities, Table 4 provides insights into load increments and cycles during the mechanical test, shedding light on the material performance under the different finishing conditions. All groups started to fail at 800 N (in between 65,000 and 75,000 cycles), with survival probabilities of 83% for the CTRL group, 75% for CCG, and 92% for C+G*.*

**Table 4:** Survival probabilities for different load steps and number of cycles.

Groups	Load/Cycles													
	200/ 5 x 10³	…	750/ 65x10³	800/ 75x10³	850/ 85x10³	900/ 95x10³	950/ 105x10³	1000/ 115x10³	1050/ 125x10³	1100/ 135x10³	1150/ 145x10³	1200/ 155x10³	1250/ 165x10³	1300/ 175x10³
CTRL	…	…	…	0.83 (0.11)	0.58 (0.14)	0.50 (0.14)	0.42 (0.14)	0.33 (0.14)	0.25 (0.13)	0.25 (0.13)	0.17 (0.11)	0.08 (0.08)	0 (0)	-
CCG	…	…	…	0.75 (0.13)	0.75 (0.13)	0.58 (0.14)	0.50 (0.14)	0.42 (0.14)	0.25 (0.13)	0.17 (0.11)	0.17 (0.11)	0.17 (0.11)	0.08 (0.08)	0 (0)
G+C	…	…	…	0.92 (0.08)	0.83 (0.11)	0.83 (011)	0.67 (0.14)	0.42 (0.14)	0.42 (0.14)	0.42 (0.14)	0.25 (0.13)	0.17 (0.11)	0.17	0 (0)

- The symbol "-" indicates the absence of a specimen being tested on the considered step.

- The symbol "…" indicates the absence of specimen fracturing in the respective step for each condition.

- Survival probabilities and their respective standard error measurements considering the progression of steps during fatigue testing (load and number of cycles for failure).

The Weibull modulus indicated no differences between groups (Table 3), showing that glazing application did not affect the mechanical reliability, considering the evaluated parameters.

**Figure 1:**
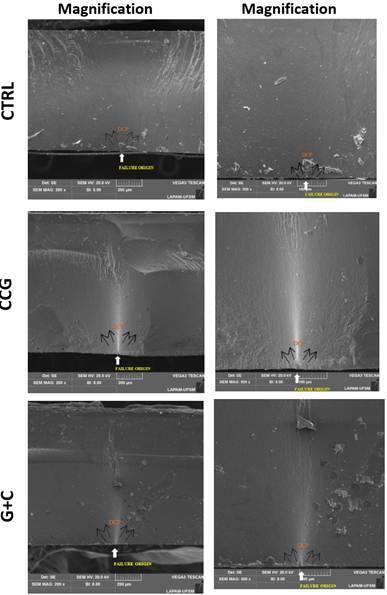
SEM images reveal radial cracks initiating at the cementation surface under tensile stress in all groups. Filled arrows mark the origin; dashed arrows indicate the direction of crack propagation (DCP).

The SEM fractographic analysis revealed a consistent failure mode in all groups, with cracks initiating at the cementation surface under tensile stress and propagating toward the top surface (Figure 1).

## Discussion

According to the findings of the present study, the glaze application protocol influenced the surface roughness of the evaluated lithium disilicate restorative materials, thus rejecting the first null hypothesis, pointing out that the additional step involved for glazing had a negative impact on Ra.

Surface roughness is a factor that influences material properties, such as wear resistance, fatigue resistance, and esthetics^25^. In this sense, glaze application is employed in the context of dental restoration manufacturing to enhance surface quality and create a glossy outer layer. Jurado et al. (2023)^26^ found that both glaze and polishing significantly reduced the surface roughness (Ra) of milled crowns of CEREC Tessera, when compared to no polishing or glazing. Such results indicate that either glaze or polishing should be performed for finishing restorations after milling^27^. However, another study showed that when comparing both conditions, glazed surfaces may exhibit a higher tendency for bacterial attachment and oral biofilm formation^28^, particularly when the recommended threshold of 0.2 μm for Ra surface roughness is not maintained^29^. In our study, polished and crystallized CEREC Tessera (CTRL group) resulted in a Ra value of 0.04 μm, well below this threshold. Two additional studies support our findings, in which Ra was analyzed on the surface of CEREC Tessera discs after polishing and crystallization, and the roughness values were very close to ours^18^ ^,^ ^30^. Lu et al. (2023)^18^ also observed an increase in roughness for glazing groups after spray glaze application, both for the combined glazing/crystallization firing and two-step firings (crystallization followed by glazing), thus corroborating our findings and indicating that spray glaze application may not generate homogenous surfaces as those of polished restorations^25^, resulting in higher values of Ra for CCG, C+G groups (Table 3)^31^. 

The optical behavior of CEREC Tessera was evaluated under different firing conditions, including the application of a glaze spray layer. No statistically significant differences were found among the tested groups, thus confirming the second null hypothesis. Achieving a tooth-like appearance is essential for esthetic dental restorations, as greater light transmission through the material contributes to a more natural result^32^. In glass-ceramics, the optical properties are closely related to the heat-treatment temperature, which influences crystal nucleation and growth^33^. Within the limits of this study, the firing conditions applied did not alter these processes sufficiently to change the optical outcome. According to the perceptibility threshold proposed by Lee (2015)^23^ (ΔTP > 2), no visible difference was detected between glazed and polished specimens. From a clinical standpoint, this finding suggests that different glaze firing protocols may be selected based on convenience rather than esthetic impact. Moreover, because no previous studies have compared these firing strategies for this ceramic, the present research provides early reference data to guide future investigations.

Fatigue loading is one of the leading causes of clinical restoration failure, characterized by progressive crack propagation under cyclic stresses^34^. In the present study, the fatigue failure load (FFL) values were statistically comparable among all groups, indicating that the glaze application protocol did not significantly influence fatigue resistance. Therefore, the third null hypothesis was accepted.

According to the manufacturer, an additional glaze firing (matrix firing) after crystallization, at 760°C for 4.5 to 12 minutes, is recommended to enhance the material’s mechanical properties^35^. Lu et al. (2023)^18^ reported that an additional glaze firing can improve flexural strength when compared to a crystallization-only firing. In our fatigue testing, however, no significant improvement in fatigue behavior was observed for the groups subjected to glaze firing (C+G and CCG) when compared to the crystallized-only group (CTRL). This result may be explained by the stress concentration during fatigue loading. This test configuration allowed fractures to consistently initiate at the cementation surface under loading (Figure 1), indicating critical tensile stress far from the glaze layer, which was applied on the top surface. Therefore, the microstructural changes promoted by the glaze firing likely occurred in a region not critically involved in the crack initiation process. Furthermore, the temperature of the additional firing did not significantly affect the strength at the cementation interface, where tensile stresses concentrate. 

In addition to fatigue failure loads, the Weibull modulus was calculated to assess the mechanical reliability of each group. The values ranged from 6.44 to 7.41, with overlapping confidence intervals for all groups, indicating comparable reliability among the tested protocols. These results suggest that glaze application did not influence the consistency of failure under fatigue loading, which is consistent with the SEM observations showing similar crack initiation patterns at the cementation interface.

Despite the findings of this study, certain limitations should be acknowledged. As an in vitro investigation, the applied load conditions and specimen geometry do not fully replicate those encountered in the clinical setting. Moreover, the evaluation was limited to two glazing protocols, which restricts broader generalization and introduces uncertainties regarding the performance of CEREC Tessera under alternative or extended firing cycles. Future studies are therefore encouraged to investigate different thermal protocols and durations to elucidate further their effects on the mechanical and optical properties of this material. The present findings nevertheless provide meaningful insights for clinicians aiming to optimize surface finishing strategies and enhance the long-term performance of lithium disilicate restorations in daily clinical practice.

Considering the limitations of this study, it can be concluded that there was a notable difference in surface roughness parameters (Ra and Rz) among the groups, with glazed specimens (CCG and C+G) exhibiting a rougher surface compared to non-glazed and only crystallized CEREC Tessera. However, the glazing application protocol, whether combined with or subsequent to crystallization, showed no detrimental or beneficial effects on the translucency and fatigue behavior of CEREC Tessera.

## Data Availability

The research data are available upon request.
